# Supplementary material for: Assessment of environmental variability on malaria transmission in a malaria-endemic rural dry zone locality of Sri Lanka: The wavelet approach
Source: PLoS One. 2020 Feb 21;15(2):e0228540. doi: 10.1371/journal.pone.0228540 (PMC7034797; doi:10.1371/journal.pone.0228540)
Supplement: Attachment — Submitted filename: Response to reviewers 13.12.2019.docx [file pone.0228540.s001.docx]

**Response to Additional Editor’s Comment**

We are very thankful for the constructive comment made, which helps to improve the manuscript. We have addressed the comment below;

1. “Thank you for clarifying that the data underlying this study are third party data. Before we can proceed, we kindly ask the authors to:”
   a. Explain how others can access these datasets

**These third party datasets will be obtained upon submitting a formal request to head of the following relevant authorities:**

i. **Malaria Research Unit of University of Colombo (**[**http://med.cmb.ac.lk/**](http://med.cmb.ac.lk/)**) - Malaria cases in Kataragama Medical Officer of Health area (free of charge)**
ii. **Department of Irrigation (**[**http://www.irrigation.gov.lk/**](http://www.irrigation.gov.lk/)**) - Data of rainfall and water flow of ‘Menik ganga’ river (free of charge)**
iii. **Department of Meteorology (**[**http://www.meteo.gov.lk/index.php?lang=en**](http://www.meteo.gov.lk/index.php?lang=en)**)** - **Data of temperature such as mean temperature, minimum and maximum temperature (on a nominal charge)**
b. “Confirm that others would be able to access these data in the same manner as the authors. Please also confirm that the authors did not have any special access privileges that others would not have.”

**Authors confirm that others would be able to access these data in the same manner as the authors and also confirm that we did not have any special access privileges that others would not have.**

2. Please ensure that you include a title page within your main document. You should list all authors and all affiliations as per our author instructions and clearly indicate the corresponding author.

**A title page has been attached as a separate word document which will be considered within the main pdf document. It includes a list of all authors and their affiliations as per author instructions and clearly indicated the corresponding author.**

3. Please upload a copy of Figure 1-6 which you refer to in your text. Or if the figure is no longer to be included as part of the submission please remove all reference to it within the text.

**A copy of Figures 1-6 has been uploaded.**
